# Linitis Plastica in a Patient With a BRCA2 Mutation: A Case Report

**DOI:** 10.7759/cureus.102423

**Published:** 2026-01-27

**Authors:** Selina Park, Edward Cao, Surabhi Amar

**Affiliations:** 1 Medicine, Creighton University School of Medicine, Phoenix, USA; 2 Hematology-Oncology, Valleywise Health Medical Center, Phoenix, USA

**Keywords:** brca2, brca2 screening, gastric cancer, linitis plastica, metastasis

## Abstract

*BRCA2* pathogenic mutations are well-established risk factors for breast, ovarian, prostate, and pancreatic cancers. However, the relationship between *BRCA2 *mutations and gastric cancer (GC) remains understudied. This case involves a patient with a pathogenic *BRCA2 *mutation diagnosed with linitis plastica, a diffuse form of GC characterized by stiffening of the gastric lining, akin to a “leather bottle.” This case highlights the importance of further research to elucidate the connection between GC and *BRCA2 *mutations, which may lead to expanded surveillance guidelines for affected individuals. Clinicians should consider the potential elevated risk of GC in patients with *BRCA2* mutations.

## Introduction

Gastric cancer (GC) was the fifth most common cancer and the fifth leading cause of cancer-related mortality worldwide in 2022 [1]. Linitis plastica (LP) is a subtype of diffuse GC characterized by macroscopic thickening and stiffening of the gastric lining, akin to a “leather bottle” [2,3]. LP accounts for approximately 10% of GC cases and is slightly more prevalent among females and younger patients [4,5]. It typically presents with progressive symptoms of nausea, vomiting, and dyspepsia [2].

Microscopically, LP is commonly described as a poorly differentiated adenocarcinoma with diffuse submucosal spread; hence, endoscopy and biopsy may fail to detect these abnormalities in the early stages [3,5,6]. Patients with the LP subtype of GC have significantly lower overall survival rates than those with non-LP subtypes [5]. Approximately 69% of LP patients are diagnosed at stage IV [6]. Current treatment for LP includes surgical resection and systemic therapy such as chemotherapy and radiation [5]. However, the mean overall survival remains limited at about 28.4 months despite treatment [6].

LP demonstrates considerable molecular heterogeneity and is associated with various genetic mutations [4]. Somatic mutations in *CDH1* have been strongly associated with LP, as *CDH1* encodes E-cadherin, a protein essential for cell-to-cell adhesion [7]. Germline *CDH1* mutations are linked to an increased risk of hereditary diffuse GC, and GC surveillance guidelines are available for affected individuals [8]. Mutations in *MUC6*, which encodes mucins that contribute to the protective gastric mucus layer, are also frequently found in LP patients [7,9]. Many of the genes mutated in LP are noted to act as tumor suppressors in the Hippo signaling pathway, a critical regulator of cellular proliferation and tissue fibrosis. Thus, dysfunction of the Hippo pathway may be a key step in the pathogenesis of LP [7].

Several hereditary syndromes are associated with increased GC risk, including Lynch syndrome (*MLH1*, *MSH2*), Peutz-Jeghers syndrome (*STK11*), and juvenile polyposis syndrome (*SMAD4*), all of which have established endoscopic surveillance guidelines in place [10]. GC has also been associated with somatic alterations in *TP53*, *KRAS*, *ARID1A*, *PIK3CA*, *ERBB3*, and *PTEN* [11]. These findings highlight the genetic heterogeneity of GC and the importance of identifying additional genetic factors that may influence GC susceptibility.

*BRCA2*, located on chromosome 13q12.3, is a tumor suppressor gene essential for homologous recombination through the promotion of RAD51-mediated repair of double-strand DNA breaks [12]. Loss-of-function pathogenic *BRCA2* mutations disrupt this pathway, resulting in genomic instability and increased predisposition to cancer [12]. Although *BRCA2* mutations are well-established risk factors for breast, ovarian, prostate, and pancreatic cancers, there is currently no literature supporting a specific association between *BRCA* germline mutations and the pathogenesis of LP [13]. Here, we describe a unique presentation of LP in a patient with a pathogenic *BRCA2* germline mutation.

This article was previously presented as a poster at the 2025 Society of General Internal Medicine Southwest Regional Meeting on January 25, 2025.

## Case presentation

The patient was a 40-year-old female who presented with a few weeks’ history of epigastric pain and abdominal bloating, limiting her ability to eat. Her medical history was notable for gastroesophageal reflux disease (GERD), but the abdominal pain was described as different from her usual GERD-associated pain. Her family history was significant for a mother with a germline *BRCA2* mutation-related ovarian cancer, both grandmothers with colon cancer, and a maternal uncle with renal cancer.

**Figure 1:**
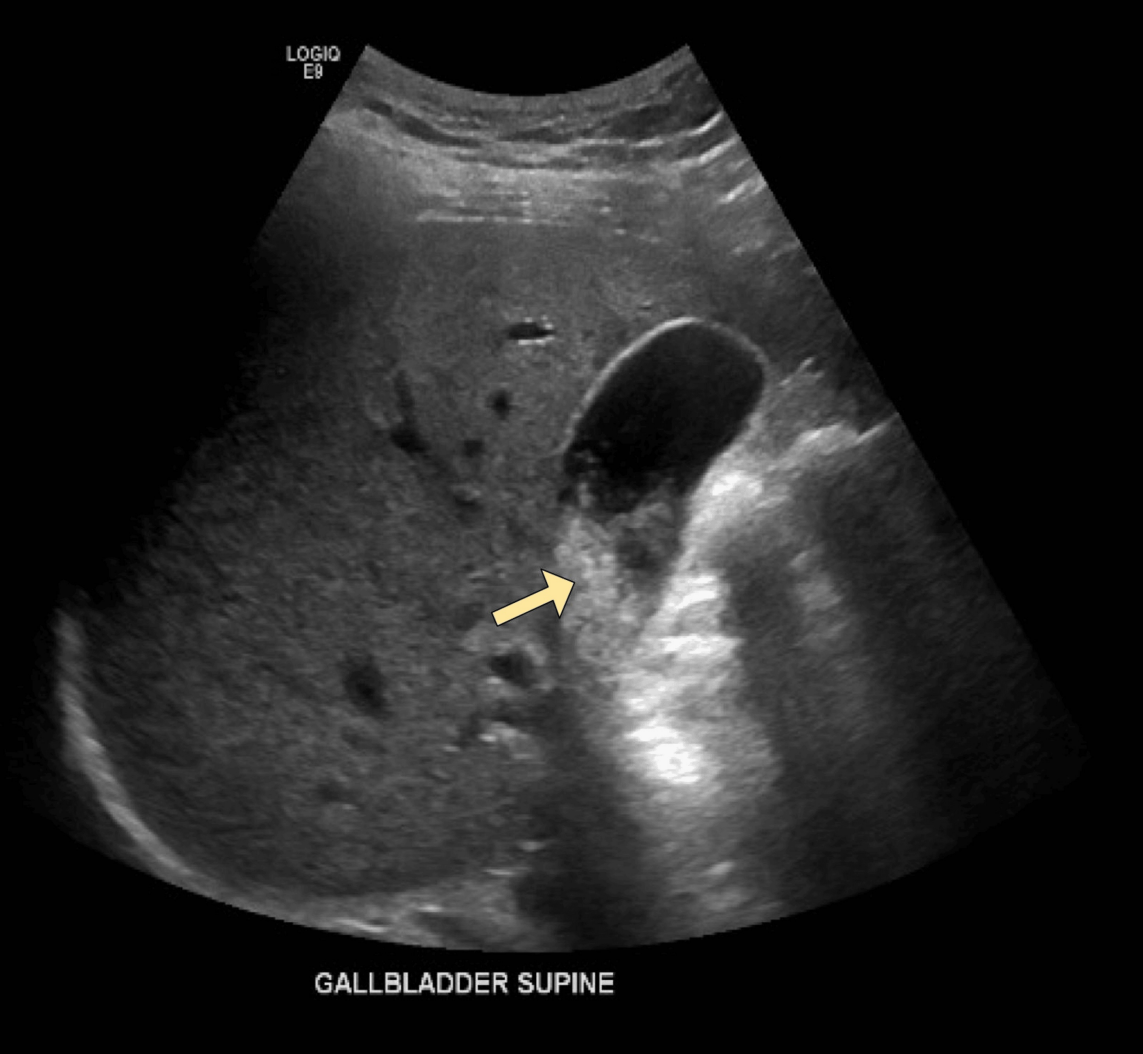
Abdominal US performed at initial presentation showing cholelithiasis (arrow) without evidence of acute cholecystitis US: ultrasound

On physical exam, the patient’s abdomen was soft and non-distended but tender in the epigastric and right upper quadrant areas. Lipase was elevated at 874 U/L (normal range: 23-300 U/L), but complete blood count (CBC) and complete metabolic panel (CMP) were otherwise unremarkable. Abdominal ultrasound (US) revealed cholelithiasis without evidence of acute cholecystitis (Figure 1). Based on the elevated lipase levels and history of epigastric pain, the patient was diagnosed with acute pancreatitis. However, the patient left against medical advice.

**Figure 2:**
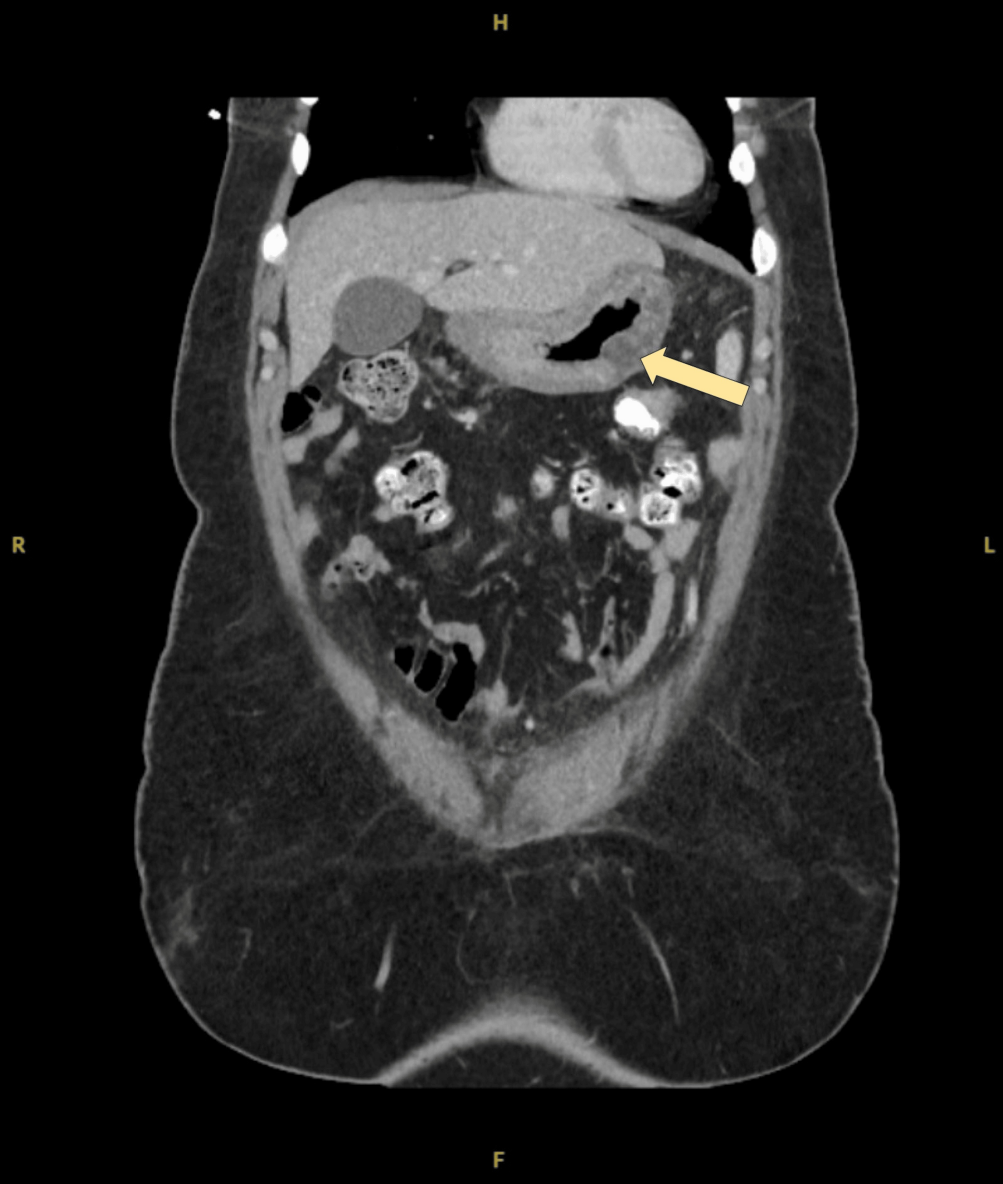
CT of the abdomen and pelvis (coronal view) demonstrating diffuse gastric mucosa thickening and a hypodense mass (arrow) within the greater curvature of the stomach CT: computed tomography

Three days later, the patient returned to the emergency room with persistent abdominal pain, nausea, and non-bloody vomiting. Lipase had decreased to 614 U/L. Repeat CBC and CMP were within normal limits. Computed tomography (CT) of the abdomen and pelvis revealed diffuse gastric mucosa thickening, a 5.3 × 1.8 × 4.2 cm hypodense mass within the greater curvature of the stomach, perigastric lymphadenopathy, and small-volume ascites, raising concerns for a gastric malignancy with peritoneal carcinomatosis (Figure 2).

**Figure 3:**
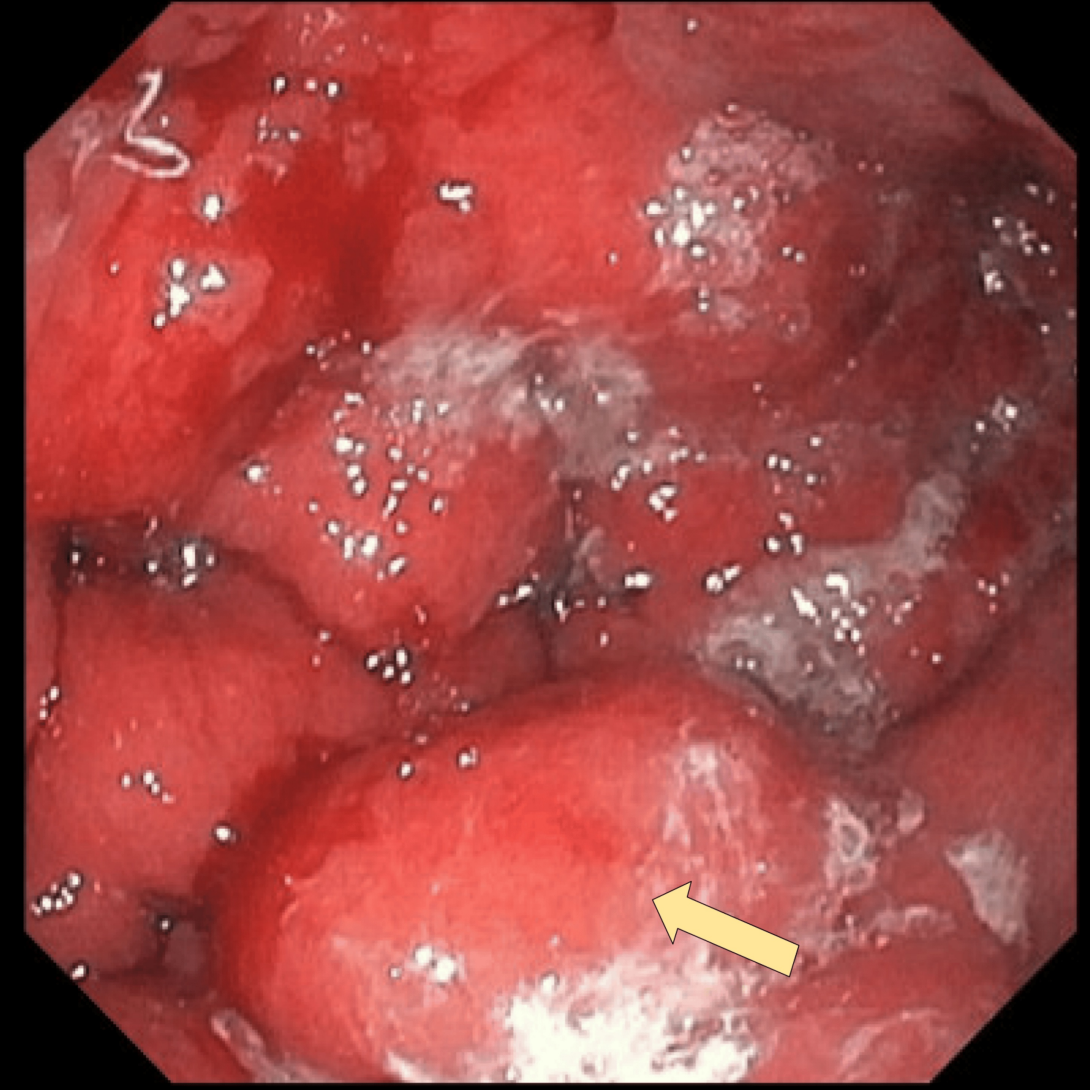
Upper endoscopy demonstrating firm, enlarged gastric folds (arrow) in the body of the stomach, concerning for LP LP: linitis plastica

**Figure 4:**
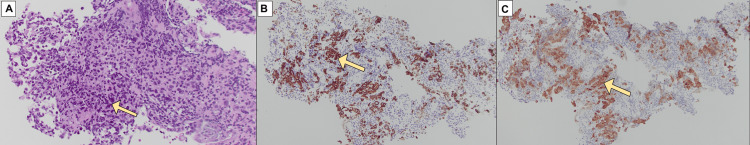
Poorly differentiated, diffuse-type gastric adenocarcinoma on histopathological examination (10x magnification) (A) Hematoxylin and eosin stain showing diffusely scattered malignant cells with enlarged, dark nuclei (arrow). (B) Pancytokeratin stain showing cytoplasmic positivity in malignant cells, confirming epithelial origin (arrow). (C) Villin stain showing cytoplasmic positivity in malignant cells (arrow).

An upper endoscopy revealed erythematous, firm, enlarged gastric folds suspicious for LP (Figure 3). A full endoscopic exam could not be completed because even a pediatric endoscope could not traverse into the duodenum due to severe pyloric stenosis. Histopathological examination of the gastric biopsy confirmed a poorly differentiated, diffuse-type adenocarcinoma consistent with primary gastric malignancy (Figure 4A-4C). Paracentesis was performed, and cytology was positive for poorly differentiated adenocarcinoma, establishing a diagnosis of stage IV GC.

Next-generation sequencing of the tumor was performed using a plasma cell-free DNA assay (Guardant360). This identified a *BRCA2* mutation with a variant allele frequency (VAF) of 47.6%, suggesting a germline mutation. Additional somatic alterations detected included *TP53* (VAF 1.9%) and *APC* (VAF 0.2%) (Table 1). Follow-up germline genetic testing confirmed a pathogenic germline *BRCA2* mutation.

**Table 1:** Next-generation sequencing results showing detected tumor alterations DNA: deoxyribonucleic acid

Detected alterations/biomarkers	% circulating free DNA or amplification
*BRCA2* C3233Wfs*15	47.6%
*TP53* C275G	1.9%
*APC* K86*	0.2%

The patient underwent jejunostomy tube (J-tube) placement to palliate symptoms of pyloric stenosis. Inpatient chemotherapy was deferred due to nutritional instability. Subsequently, palliative chemotherapy with 5-fluorouracil, leucovorin, oxaliplatin, and docetaxel (FLOT regimen) was initiated.

## Discussion

Beyond the well-established cancer risks associated with *BRCA2* mutations, emerging evidence demonstrates an increased risk of GC among *BRCA2* carriers. A 2022 multi-institutional case-control study showed that pathogenic variants in *BRCA2* are associated with an increased risk of GC, with a reported lifetime cumulative risk of 19.3%. Of note, the study was conducted in Japan, where the baseline incidence of GC is higher than in the United States [14]. A 2021 meta-analysis involving studies from multiple countries showed that *BRCA2* mutations increase GC risk, with a relative risk of 2.15 [15]. Nevertheless, an association between *BRCA2* mutations and LP has not been reported in the current literature.

The *BRCA2* C3233Wfs*15 variant identified in this case involves a frameshift mutation that produces a truncated, nonfunctional protein lacking nuclear localization signals [16]. The disruption of homologous recombination and impaired DNA repair, leading to genomic instability, may have contributed to the development of LP in this patient.

While the National Comprehensive Cancer Network has established screening guidelines for *BRCA2* mutation-associated breast, ovarian, prostate, and pancreatic cancers, no such guidelines exist for GC [17]. The probable reasons for the lack of screening guidelines are the relatively small sample size of GC cases and the absence of a clear mechanistic link between *BRCA2* mutations and GC development. Establishing a definitive connection between *BRCA2* mutations and GC could lead to targeted screening guidelines and reduce the incidence of advanced-stage diagnoses, improving patient prognosis.

In addition to screening, further study of *BRCA2* and its connection to LP may reveal potential benefits of *BRCA2*-targeted therapies, such as poly(adenosine diphosphate ribose) polymerase (PARP) inhibitors. These inhibitors trap PARP, a DNA repair enzyme, thereby preventing DNA repair and promoting tumor cell death. PARP inhibitors have shown efficacy in *BRCA*-mutated cancers, as these mutations already cause faulty DNA repair [18]. However, studies on the clinical benefits of PARP inhibitors in GC remain inconclusive. The phase III GOLD trial conducted in Asia did not demonstrate a significant improvement in overall survival with PARP inhibitor therapy in advanced GC [19]. However, preclinical studies suggest that GC with homologous recombination deficiency may benefit from *BRCA*-targeted therapy, including PARP inhibitors [20]. Further multinational clinical trials and meta-analyses focusing on *BRCA*-mutated or homologous recombination-deficient GC subtypes are needed to clarify the potential role of PARP inhibitors in GC treatment.

## Conclusions

This case highlights the co-occurrence of LP and a pathogenic *BRCA2* mutation, suggesting that homologous recombination deficiency may be one mechanism contributing to the pathogenesis of LP. The case also raises important questions about the possible link between *BRCA2* mutations and GC. It underscores the need to consider GC as a potential diagnosis in *BRCA2* mutation carriers presenting with gastrointestinal symptoms. Further research is essential to clarify how *BRCA2* mutations contribute to LP, thereby improving our understanding of the disease and supporting the expansion of surveillance guidelines and treatment options for this rare malignancy.
